# Assessing outcomes of ear molding therapy by health care providers and convolutional neural network

**DOI:** 10.1038/s41598-021-97310-7

**Published:** 2021-09-09

**Authors:** Rami R. Hallac, Sarah A. Jackson, Jessica Grant, Kaylyn Fisher, Sarah Scheiwe, Elizabeth Wetz, Jeyna Perez, Jeon Lee, Krishna Chitta, James R. Seaward, Alex A. Kane

**Affiliations:** 1 Department of Plastic Surgery, UT Southwestern, 5323 Harry Hines Blvd, Dallas, TX 75390 USA; 2 Analytical Imaging and Modeling Center, Children’s Medical Center, Dallas, 1935 Medical District Dr., Dallas, TX 75235 USA; 3 Department of Bioinformatics, UT Southwestern, 5323 Harry Hines Blvd, Dallas, TX 75390 USA

**Keywords:** Outcomes research, Paediatric research

## Abstract

Ear molding therapy is a nonsurgical technique to correct certain congenital auricular deformities. While the advantages of nonsurgical treatments over otoplasty are well-described, few studies have assessed aesthetic outcomes. In this study, we compared assessments of outcomes of ear molding therapy for 283 ears by experienced healthcare providers and a previously developed deep learning CNN model. 2D photographs of ears were obtained as a standard of care in our onsite photography studio. Physician assistants (PAs) rated the photographs using a 5-point Likert scale ranging from 1(poor) to 5(excellent) and the CNN assessment was categorical, classifying each photo as either “normal” or “deformed”. On average, the PAs classified 75.6% of photographs as good to excellent outcomes (scores 4 and 5). Similarly, the CNN classified 75.3% of the photographs as normal. The inter-rater agreement between the PAs ranged between 72 and 81%, while there was a 69.6% agreement between the machine model and the inter-rater majority agreement between at least two PAs (i.e., when at least two PAs gave a simultaneous score < 4 or ≥ 4). This study shows that noninvasive ear molding therapy has excellent outcomes in general. In addition, it indicates that with further training and validation, machine learning techniques, like CNN, have the capability to accurately mimic provider assessment while removing the subjectivity of human evaluation making it a robust tool for ear deformity identification and outcome evaluation.

## Introduction

Congenital ear deformities in newborns present along a spectrum of severity and may negatively affect a patient’s quality of life when left untreated. Examples of ear deformities treatable by molding include diagnoses such as Stahl’s ear, cup ear, cryptotia, helical rim deformities, lidding/lop ear, and prominent ears^1^. These deformities, often a result of external forces on normal ears like malposition while sleeping or pressure from the birth canal, occur with an incidence between 6 and 58%^2^. Although an estimated 30% of these cases will self-correct, most deformed newborn ears benefit from medical intervention^2^.

Traditionally, congenital ear deformities are treated with surgical correction after the age of 5 years, or once the ear has reached closer to full size, and when the cartilage firms to the point where it will hold suture. Recently, nonsurgical techniques have been used to correct ear deformity, such as splinting and ear molding^3^. These techniques are noninvasive, painless, and avoid morbidities that can be associated with traditional otoplasty^4,5^. The range of deformities that are treatable with neonatal molding is distinct from those congenital deformities that result from hypoplasia of skin or cartilage, which generally require surgical correction^6,7^. While ear molding has exhibited a high level of efficacy, it has yet to achieve widespread use due to a lack of recognition, delays in proper diagnosis, or an incorrect assumption that most ear deformities will self-correct. Often, by the time an appropriate diagnosis has been made, the window for ear molding has passed^2,8^. Timing is essential for this nonsurgical treatment because it utilizes the malleable auricle cartilage that is present from birth up until 6 weeks of life^3^. This advantageous malleability is a result of high levels of circulating maternal estrogen, which reaches its peak during delivery. Maternal estrogen increases levels of hyaluronic acid, causing the cartilage to become more pliable and amenable to molding from external forces. Circulating maternal estrogen begins to decline in the infant at 3 days of age up until around 6 weeks of age. With the depletion of maternal estrogen in the infant, the ears become much less pliable, therefore rejecting molding as a viable treatment option^2^. Therefore, it is imperative that a swift and accurate diagnosis is made early in the infant’s life.

While the advantages of nonsurgical treatments are evident, few studies have assessed their aesthetic outcomes. Current clinical assessment relies on subjective opinion, such as expert ratings^1,6,9^. Due to the complex ear structure, it can be difficult to ensure a standard is kept among providers and, therefore, there is currently no gold standard to evaluate treatment outcomes. In this study, we aimed to assess our noninvasive ear molding technique using Likert-scale survey and a convolutional neural network (CNN) model previously developed by our center.

CNN is a deep learning method that has had great success in medical image analysis to classify and analyze diseases and is a promising solution to assess ear deformity and treatment outcomes^5^. In addition, its application has been successful in ear recognition^10–12^ and has shown to be superior to traditional computer vision systems that uses feature extraction algorithm such as principal component analysis, speeded up robust features^11^, or even handcrafted features^13^. The success of CNN can be attributed to its deep layer structure and has shown to excel in image analysis. Recently, several studies have developed visualization tools to demystify the complexity of deep networks and to help understand the importance of each neuron to target decision^14–17^. These tools, such as Gradient-weighted class activation mapping (Grad-CAM), highlight significant regions in the input image that affected the CNN predictions^16,18^. Visualization tools have been used in ear recognition studies and have increased the trust and reliability of the CNN prediction by providing comprehensive analysis^16^. In a previous study, we developed a CNN model to extract features from 2D photographs of ears and classify each image as normal or abnormal. The CNN model was able to identify ear abnormalities with a high accuracy (94.1%)^5^. The effectiveness of this CNN model to classify abnormality suggests that it can potentially be used for diagnosis and objective medical assessment.

This study describes the effectiveness of nonsurgical ear molding treatment utilized at our center by three expert providers and our CNN model.

## Results

### Molding treatment

In this study, we evaluated 283 ears from 155 individual patients (40% female). 82.6% of the patients had bilateral ear correction. Most patients (74.2%) started ear molding therapy before 14 days of age. 83.9% of patients completed their treatment within 6 weeks, and 16.1% of patients completed their treatment within 12 weeks. Treatment was applied to the following ear deformities (Table 1): lidding (47.3%), helical kinks (18.0%), Stahl's (12.0%), constricted ear (8.5%), prominent ear (6.4%), satyr (6%), and cryptotia (1.8%).

**Table 1:** Patient demographics and deformity information.

Characteristic	Count (%)
Patient count	155
Ear count	283
**Sex**	
Male	93 (60.0%)
Female	62 (40.0%)
**Ear deformity**	
Unilateral	27 (17.4%)
Bilateral	128 (82.6%)
**Subtype**	
Lidding	134 (47.3%)
Kinking	51 (18.0%)
Stahl's	34 (12.0%)
Constricted	24 (8.5%)
Prominent	18 (6.4%)
Satyr	17 (6.0%)
Cryptotia	5 (1.8%)
**Age at initiation of therapy**	
< 14 days of age	210 (74.2%)
> 14 days of age	73 (25.8%)
**Treatment length**	
0–2 weeks	8 (5.2%)
2–4 weeks	61 (39.4%)
4–6 weeks	61 (39.4%)
6–8 weeks	13 (8.4%)
8–10 weeks	8 (5.2%)
10–12 weeks	4 (2.6%)

### Physician assistants’ ratings

When analyzing the PA scores, all ratings of 4 or 5 were considered “good outcomes” and all ratings of 1, 2, or 3 were considered to exhibit some degree of residual deformity. The overall mean score for all PAs was 4.0 ± 0.9. On average, the PAs rated 75% of the photographs a 4 or 5.

Table 2 show the distribution of scores for each individual PA. PA 1 rated 87.6% of the ears above 4, with an average score of 4.2, which is higher than PA 2 and PA 3 with 75.3% and 64.0% of the ears above 4 (mean scores 4 and 3.8, respectively).

**Table 2:** The distribution of scores for each individual PA.

	PA1		PA2		PA3	
	Count	%	Count	%	Count	%
Rating of 1	0	0.0	2	0.7	5	1.8
Rating of 2	9	3.2	7	2.5	33	11.7
Rating of 3	26	9.2	61	21.6	64	22.6
Rating of 4	138	48.8	132	46.6	90	31.8
Rating of 5	110	38.9	81	28.6	91	32.2
% of 4 and 5 scores (normal)		87.6		75.3		64.0

**Figure 1:**
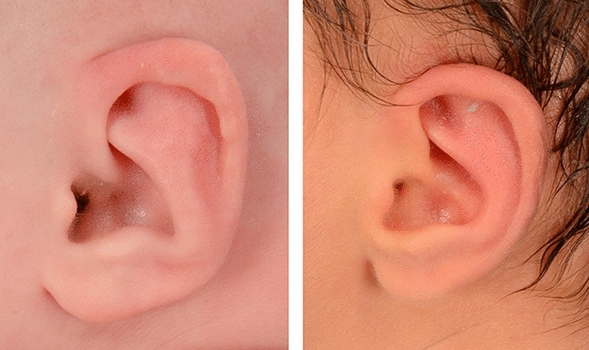
Post therapy outcome of two ears rated 3 (left) and 5 (right) by all PAs.

In general, there was agreement among the three PAs. PA 1 and PA 2 agreed 80.1% when they rated the 2D photographs. A chi-square test of independence showed the relation between these variables was significant, X^2^ = 46.8, *p* < 0.0001. Similarly, there was 72% agreement between PA 1 and PA 3 (X^2^ = 38.0, *p* =  < 0.0001) and 81% agreement between PA 2 and PA 3 (X^2^ = 99.5, *p* =  < 0.0001). Figure 1 shows an example of therapy outcomes rated 5 (right) and 3 (left) by all PAs.

### Deep learning assessment

**Figure 2:**
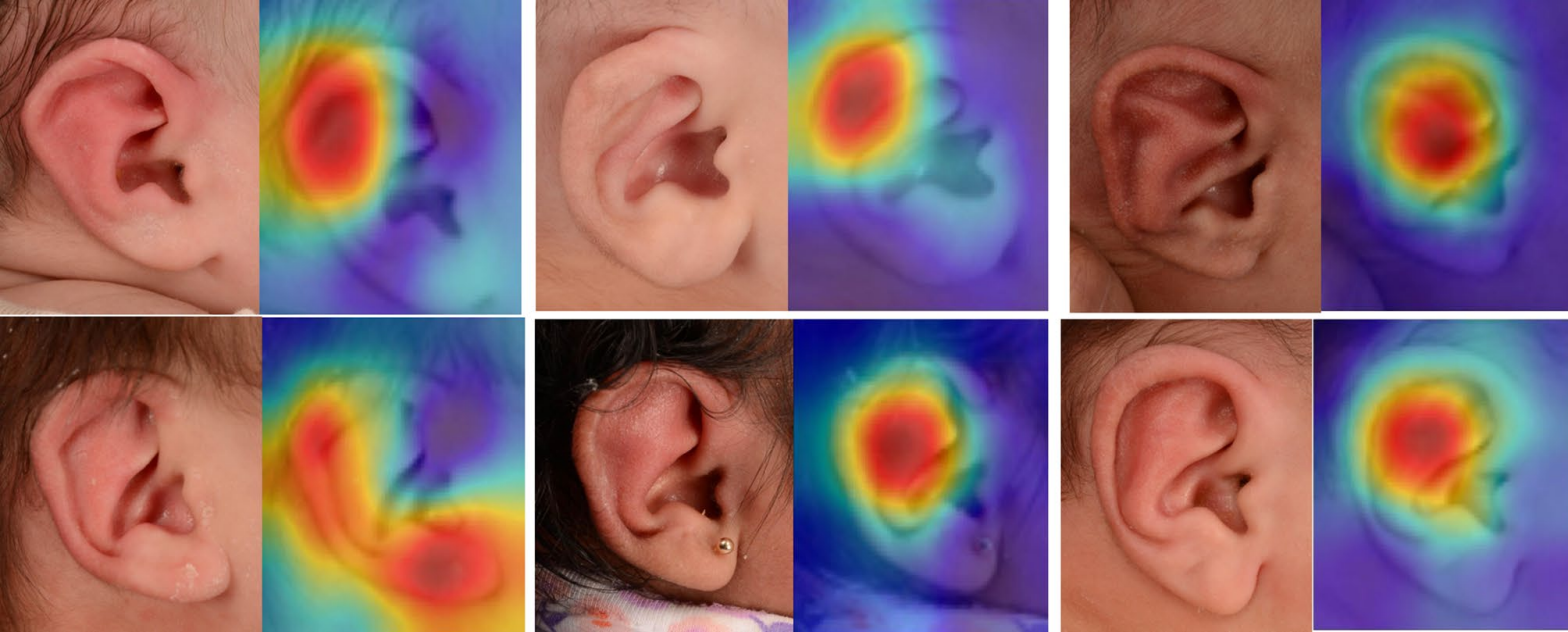
Top row: ears classified as deformed by CNN which are in agreement with the PAs’ evaluations (score < 4). Bottom row: ears classified as deformed by CNN but received good scores from the PAs (≥ 4), exhibiting disagreement.

**Figure 3:**
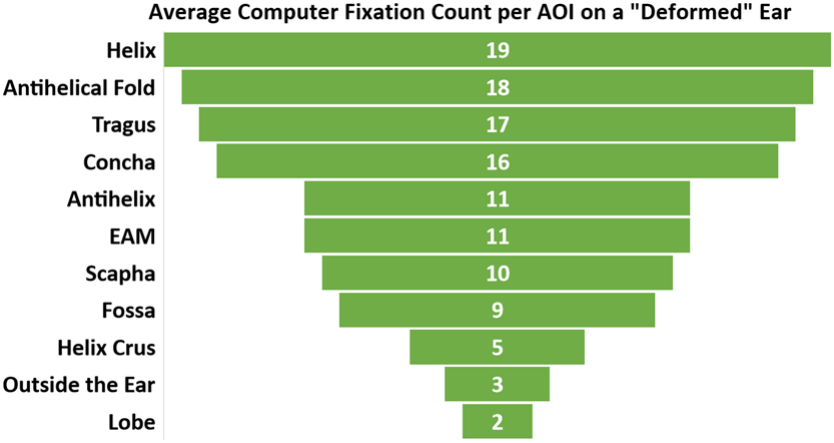
AOI that the machine used in determining the classification of ears based on Grad-Cam examination.

In agreement with the overall PA score, the CNN model classified 75.3% of the photographs as “normal” (i.e. good outcome) and 24.7% of the photographs as “deformed”. Upon visual examination of the Grad-Cam heatmaps for the images labeled as “deformed” (Fig. 2), we noticed that the machine model highlighted the majority of abnormalities in the regions of the helix (n = 19) and antihelical fold (n = 18), and the least in the region of the helix crust (n = 5) and lobule (n = 2). In three images (n = 3), the machine model mistakenly detected deformities outside the ear. Figure 3 shows the distribution of regions indicated by the machine model based on Grad-Cam examination.

**Figure 4:**
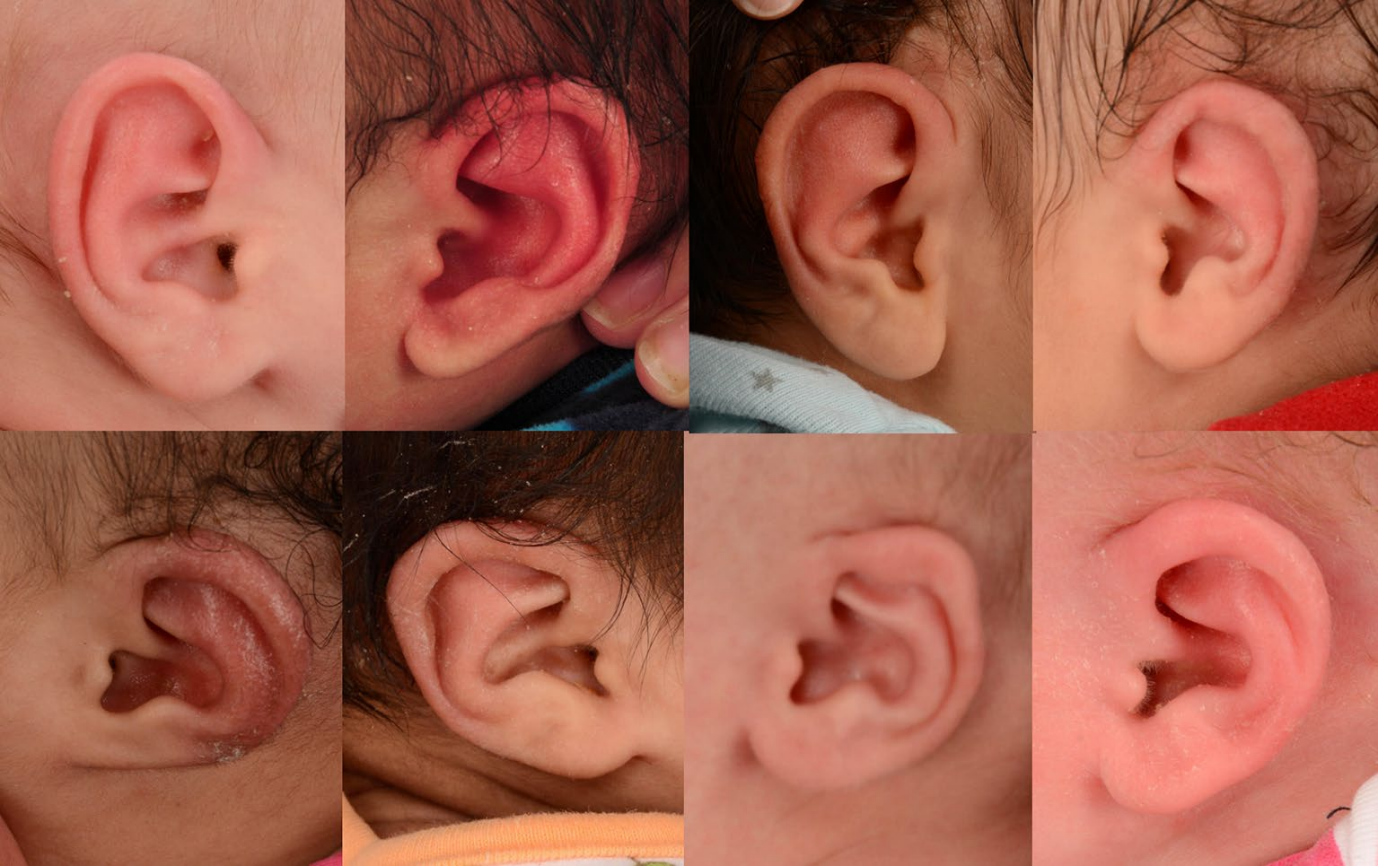
Top row: ears classified as normal by both PA and CNN evaluations, exhibiting agreement. Bottom row: ears classified as “normal” by the CNN model, which disagreed with the PA evaluations of deformed.

There was 69.6% agreement between the machine model and the inter-rater majority agreement between at least two PAs (i.e., when at least two PAs gave a simultaneous score < 4 or ≥ 4). A chi-square statistical test indicated a statistically significant agreement between the PAs and CNN (X^2^ = 7.2, *p* = 0.007135). Figure 4 shows examples where the machine models disagreed with the PAs’ consensus agreement.

## Discussion

This study assessed ear molding therapy for infants with ear deformity by three expert practicing PAs in applying ear molding and a previously developed CNN model. The PA scores and CNN model indicated that at least 75% of the ears were normalized after the treatment. With high rates of ear deformity among newborns, this non-invasive and inexpensive technique can provide alternative option for parents to avoid expensive, often challenging, surgical approaches.

In addition to the ear molding technique summarized in this study, other medical centers have also reported successful outcomes with various noninvasive therapies^19,20^. Ear splint technique utilizes a mechanical device that has been approved by US Food and Drug Administration as a class I medical device to hold the pinna to correct ear deformity. Woo et al.^9^ examined ear splint therapy in 41 children with mean age of 52.9 days. The treatment duration ranged between 8 to 169 days. Based on the ratings of physicians and caregivers, the authors reported significant improvement to the ear shape when compared to its initial severity^9^. Similarly, Byrd et al.^7^ reviewed over 831 ear deformities (488 patients) after therapy using an ear molding device (EarWell) and reported 90% success (good or excellent results). The authors also reported that treatment was most successful when begun at early age of life (first week). Our ear mold technique achieved 75% successful results when assessed by experienced PAs.

To date, there is no reliable objective method for the assessment of ear deformities, and most previous studies relied on subjective assessment^4,6,21^. As stated previously, without an objective diagnostic tool, ear outcome evaluations are prone to subjectivity which can vary between providers^22,23^. This can be seen in our study where the inter-rater agreement among the PAs ranged between 72 and 81%. This may be attributed to subjectivity that is inherent to human evaluation, especially when there is no gold standard for ear therapy assessment. In this study, we applied machine learning to assess ear molding therapy, which is the first objective tool and use of CNN to examine ear deformation^5,9^. Overall, about 75% of the images were classified as normal by the CNN model, which agrees with the PAs’ overall rating.

In addition, Chi square test indicated agreement (69.6% agreement, X^2^ = 7.2, *p* = 0.007135) between CNN and inter-rater majority agreement between at least two PAs (i.e., when at least two PAs gave a simultaneous score < 4 or ≥ 4). Meanwhile, when the CNN model was compared with the consensus agreement among all the PAs (i.e. when all three PAs provided a simultaneous score < 4 or ≥ 4), there was 76.3% agreement. This promising result indicates that the CNN model can be a useful tool to mimic the diagnosis and evaluation of trained providers.

Figures 2 and 3 illustrate the AOIs the CNN fixated on when classifying the ears as deformed. The most fixated area was the helix closely followed by the antihelical fold. The least fixated area was the lobe. Ear molding treatments often focus on sculpting the outer portion of the ear, specifically the helix and antihelical rim, which are affected by the most common ear deformities^2,7^. We generated Grad-CAM visualizations to obtain an insight into the process of misclassified photographs. Analysis of the Grad-CAM heatmaps indicated that the CNN model is evaluating the most vital areas of the ear. There were also areas of the ear which the CNN focused on that are not treated as frequently using ear molding including the tragus. This could indicate that ear deformity of the helix can also affect other anatomical structures of the ear. Unfortunately, there were a few erroneous cases (n = 3) where the CNN analyzed areas outside of the ear. One limitation is that this study’s neural network was trained to perform binary classification of ears as “normal” or “abnormal” and was not trained to a specific pathology with respect to the location on the auricle. We believe that training a CNN model on different types of ear deformities, spectrum of severity, or therapy outcomes will further improve the CNN accuracy. With larger annotated data, the model can be trained to classify the ears into specific pathology (e.g. lobule deformity, prominent ears, ear clefts, cup ear deformities, etc.) and, therefore, enhancing the reliability and accuracy of the CNN model. In addition, our study is limited by the number of PAs (n = 3) who evaluated the ear outcomes. Given the subjectivity in rating ear deformity outcomes, more ratings are needed to create a gold standard of outcomes. A future study might require a multicenter collaboration to obtain more expert ratings. In addition, obtaining laypersons’ rating can provide insight on whether these deformities are noticeable to the perception or awareness of laypersons.

The photographs used in this study were taken in our plastic and reconstructive surgery photography studio. While they exhibit variability in factors such as angle and zoom, most of the photographs were obtained in a professional studio with similar lighting according to standard clinical protocols. Therefore, it is important to train and test the CNN models with data obtained from outside our institution to increase the reliability of the CNN model, especially when used as smartphone applications.

Early detection and diagnosis of ear abnormality is crucial since ear molding takes advantage of the plasticity of auricular cartilage during the early neonatal period^24^. In our center, over 114 (74%) patients began treatment within the first two weeks of life, which could have contributed to the success of the treatment. Ear deformities are often diagnosed by our referring pediatric physicians during the first well-child exam, which usually occurs during the first week of life. To minimize delays in ear molding treatment due to insurance approvals, a claim is submitted before the patient’s first appointment. In some few cases where the claim is denied, the patients are provided with an option for out-of-pocket payment. The cost of treatment is low, compared to protocols utilizing commercially available molding systems. Even if an insurance company denies the claim as cosmetic and not a covered benefit, the out-of-pocket cost is about $300 per visit, for a total of three visits. This may account for the success in attracting patients early, but testing this hypothesis was beyond the scope of this study.

It is important to mention that only a few studies have assessed the outcomes of noninvasive ear deformity therapy. This may be due to lack of widespread knowledge of this therapy among health professionals^9^. In addition, it can be challenging for healthcare specialists to learn a new technique without hands-on experience. Therefore, for the past 4 years, our team has been holding workshops at international conferences as an educational tool to practice the skills necessary to undertake neonatal ear molding effectively and safely^25^. The course included the use of flexible 3D printed models that were digitally morphed to represent the three most common ear deformities (Stahl’s ear, lidding/lop ear, and prominent ear). The 3D models were printed in a soft rubberlike material (TangoPlus) using a Connex 3D printer (Stratasys).

Due to the limited data available at our center, the CNN model in this study classified ear molding outcomes as “normal” or “abnormal”. A granular classification with respect to location on the auricle (e.g. lobule, helix, tragus, etc.) may be feasible with larger annotated dataset. Yet, in this single center study, both experts’ ratings and CNN showed that ear molding is an effective nonsurgical intervention for the treatment of neonatal ear deformities. With further training and improvement, deep learning will provide caregivers an objective tool to assess and compare the efficacy of various nonsurgical methods. It could also help avoid extraneous treatments and ensure that treatment outcomes are equitable for all patients and families.

## Conclusion

This study showed that noninvasive ear molding was effective. In addition, it demonstrated that deep learning methods, like CNN, offers providers an objective evaluation tool which may obviate the problem of low inter-rater reliability. If validated and deemed universally accurate, this deep learning algorithm could be a dynamic evaluation tool that would allow for impartial evaluations of other ear reconstructive interventions.

## Methods

The study was approved by the Institutional Review Board (IRB) at UT Southwestern Medical Center, and it was carried out in accordance with IRB guidelines and regulations. The IRB approved a waiver of informed consent given that our study is a retrospective review (IRB # 032018-060). After obtaining IRB approval, we retrieved 2D photographs of patients treated with ear molding. Post treatment 2D photographs taken as standard of care in our plastic and reconstructive surgery photography studio were retrieved using our database search engine^26^. A total of 283 ears were included in this study (155 individual patients).

**Figure 5:**
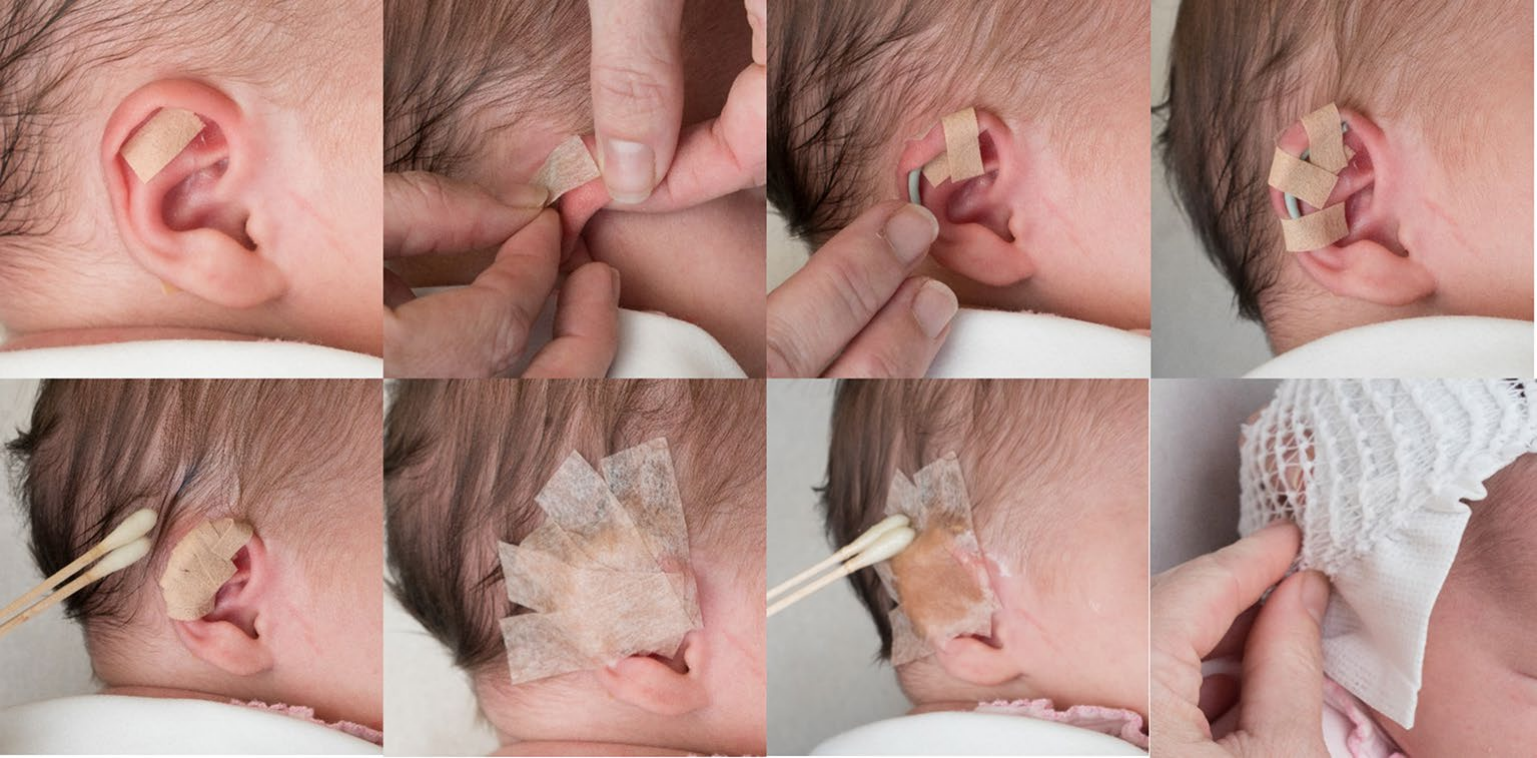
Representation of our ear molding process during treatment.

The clay ear molding treatment utilizes custom molded dental clay stents, which are secured in place on the ear using Mastisol adhesive liquid and Micropore tape (Fig. 5). Additional steri-strips or micropore tapes are placed in necessary areas to support the ear in the desired position. Ear molding is evaluated, adjusted, and reapplied every 3 weeks and is performed under the care of physician assistants (PAs) in the clinic^25^. Ear photographs were taken using a Nikon D90 with a Nikkor 24–85mmf/3.5–4.5 lens. A PocketWizard transmitter and receiver were used to trigger the strobes.

In this study, the outcomes of 283 ear molding treatments were assessed from 2D photographs by 3 experienced PAs with more than 3 years of experience providing ear molding treatment. The assessment was conducted using a 5-point Likert scale ranging from 1(poor) to 5(excellent).

In addition, the treatment outcomes were assessed using our CNN model classifying them into two categories: “normal” or “deformed”. In a previous study performed by our lab, a CNN model was trained and tested to identify ear abnormalities for 2D photographs^5^. A total of 457 photographs of deformed ears and 214 photographs of normal ears were used to train and test the CNN model developed in our previous study. Photographs were cropped to the ear boundary and randomly divided into training (60%), validation (20%), and testing (20%) datasets. We modified the SoftMax classifier in the last layer of the pretrained GoogleNet model to classify the ears as normal or abnormal. To overcome the overfitting problem that is caused by small training datasets, each training image was randomly scaled and translated per epoch. The hyperparameters used in the CNN model were as follows: batch size = 50; number of epochs = 300; and initial learning rate = 1e−4. Stochastic gradient descent with momentum was used to optimize weights during training^27^. Machine learning analyses were performed using an EVGA GeForce GTX 1080 with 8 GB onboard memory. After training the CNN model properly, it was able to achieve a 94.1% accuracy in the task of classifying ears as normal or deformed. This study utilizes the trained model to classify treatment outcomes as normal or abnormal. In addition, Gradient-weighted Class Activation Mapping (Grad-Cam) were generated over each photograph to identify important regions, which most affected the CNN model’s prediction on each photograph.

### Statistical analysis

We classified the PA ratings of 1, 2, or 3 as “average outcome” and all ratings of 4 or 5 as “good outcome”. Treatment efficacy was calculated as percentage of good outcome. In addition, comparisons between raters were made using chi-square test. The level of agreement is described as percent agreement. Data are presented as n (%).

Our CNN model assessed the same 2D photographs and placed the outcomes into one of two categories: “normal” or “deformed”. Treatment efficacy was calculated as percentage of “normal” outcomes. A majority agreement between the PA ratings was obtained to compare the agreement between the CNN and PA ratings. For instance, if PAs 1 and 2 rated the ears above 4, whereas PA 3 rated the ear below 4, the ear was considered “normal” based on majority rating. Chi-square test was used to assess the agreement between CNN and PA ratings. In all instances, *p*-values of < 0.05 were accepted as significant.

## Data Availability

Data from this study are available to interested readers upon reasonable request.

## References

[1] Feijen MMW, van Cruchten C, Payne PE, van der Hulst RRWJ (2020). Non-surgical correction of congenital ear anomalies: a review of the literature. Plast. Reconstr. Surg. Glob. Open.

[2] Chang CS, Bartlett SP (2019). Deformations of the ear and their nonsurgical correction. Clin. Pediatr. (Phila.).

[3] Schultz K, Guillen D, Maricevich RS (2017). Newborn ear deformities: early recognition and novel nonoperative techniques. Semin. Plast. Surg..

[4] Petersson RS, Recker CA, Martin JR, Driscoll CL, Friedman O (2012). Identification of congenital auricular deformities during newborn hearing screening allows for non-surgical correction: a Mayo Clinic pilot study. Int. J. Pediatr. Otorhinolaryngol..

[5] Hallac RR, Lee J, Pressler M, Seaward JR, Kane AA (2019). Identifying ear abnormality from 2D photographs using convolutional neural networks. Sci. Rep..

[6] Daniali LN (2017). Classification of newborn ear malformations and their treatment with the earwell infant ear correction system. Plast. Reconstr. Surg..

[7] Byrd HS, Langevin CJ, Ghidoni LA (2010). Ear molding in newborn infants with auricular deformities. Plast. Reconstr. Surg..

[8] Chang CS, Bartlett SP (2017). A Simplified nonsurgical method for the correction of neonatal deformational auricular anomalies. Clin. Pediatr. (Phila.).

[9] Woo JE (2017). Effectiveness of ear splint therapy for ear deformities. Ann. Rehabil. Med..

[10] Alshazly H, Linse C, Barth E, Martinetz T (2020). Deep convolutional neural networks for unconstrained ear recognition. IEEE Access.

[11] Galdámez PL, Raveane W, González Arrieta A (2017). A brief review of the ear recognition process using deep neural networks. J. Appl. Log..

[12] Tian, L. & Mu, Z. in *2016 9th International Congress on Image and Signal Processing, BioMedical Engineering and Informatics (CISP-BMEI).* 437–441.

[13] Alshazly H, Linse C, Barth E, Martinetz T (2019). Handcrafted versus CNN features for ear recognition. Symmetry.

[14] Zeiler, M. D. & Fergus, R. in *Computer Vision–ECCV 2014.* (eds David Fleet, Tomas Pajdla, Bernt Schiele, & Tinne Tuytelaars) 818–833 (Springer).

[15] Springenberg, J. T., Dosovitskiy, A., Brox, T. & Riedmiller, M. A. Striving for simplicity: the all convolutional net. *CoRR* abs/1412.6806 (2015).

[16] Alshazly H, Linse C, Barth E, Martinetz T (2019). Ensembles of Deep learning models and transfer learning for ear recognition. Sensors (Basel).

[17] Zhou, B., Khosla, A., Lapedriza, A., Oliva, A. & Torralba, A. in *2016 IEEE Conference on Computer Vision and Pattern Recognition (CVPR).* 2921–2929.

[18] Selvaraju, R. R. *et al.* in *2017 IEEE International Conference on Computer Vision (ICCV).* 618–626.

[19] Park C (2002). Correction of cryptotia using an external stretching device. Ann. Plast. Surg..

[20] Park JH, Kim KM, Lee YS, Kim YS, Kim YO (2000). Non-operative correction of congenital auricular deformities using a silicone splint. J. Korean Soc. Plast. Reconstr. Surg..

[21] Leonardi A (2012). Neonatal molding in deformational auricolar anomalies. Eur. Rev. Med. Pharmacol. Sci..

[22] Cho M-J, Hallac RR, Effendi M, Seaward JR, Kane AA (2018). Comparison of an unsupervised machine learning algorithm and surgeon diagnosis in the clinical differentiation of metopic craniosynostosis and benign metopic ridge. Sci. Rep..

[23] Cho MJ, Kane AA, Seaward JR, Hallac RR (2016). Metopic, "ridge" vs. "craniosynostosis": quantifying severity with 3D curvature analysis. J. Craniomaxillofac. Surg..

[24] Anstadt EE, Johns DN, Kwok AC-M, Siddiqi F, Gociman B (2016). Neonatal ear molding: timing and technique. Pediatrics.

[25] Wetz, E. *et al.* in *The American Cleft Palate-Craniofacial Association* Vol. 56, 1–130 (Cleft Palate Craniofac J, Tucson, Arizona, 2019).

[26] Hallac RR (2019). Digital images in academic plastic surgery: a novel and secure methodology for use in clinical practice and research. Cleft Palate Craniofac. J..

[27] Goodfellow I, Bengio Y, Courville A (2016). Deep Learning.

